# Performance of machine learning algorithms for dementia assessment: impacts of language tasks, recording media, and modalities

**DOI:** 10.1186/s12911-023-02122-6

**Published:** 2023-03-03

**Authors:** Mahboobeh (Mah) Parsapoor (Parsa), Muhammad Raisul Alam, Alex Mihailidis

**Affiliations:** 1 Centre de Recherche Informatique de Montréal (CRIM), Montreal, Canada; 2 Department of Computer Science, University of Toronto, Toronto, Canada; 3 Department Occupational Science and Occupational Therapy, University of Toronto, Toronto, Canada; 4 Vector Institute, Toronto, Canada; 5 Institute of Biomedical Engineering, University of Toronto, Toronto, Canada

**Keywords:** Dementia, Alzheimer’s disease, Mild cognitive impairment, Acoustic features, Linguistic features, Machine learning, Language impairments, Language assessment methods, Speech assessment methods

## Abstract

**Objectives:**

Automatic speech and language assessment methods (SLAMs) can help clinicians assess speech and language impairments associated with dementia in older adults. The basis of any automatic SLAMs is a machine learning (ML) classifier that is trained on participants’ speech and language. However, language tasks, recording media, and modalities impact the performance of ML classifiers. Thus, this research has focused on evaluating the effects of the above-mentioned factors on the performance of ML classifiers that can be used for dementia assessment.

**Methodology:**

Our methodology includes the following steps: (1) Collecting speech and language datasets from patients and healthy controls; (2) Using feature engineering methods which include feature extraction methods to extract linguistic and acoustic features and feature selection methods to select most informative features; (3) Training different ML classifiers; and (4) Evaluating the performance of ML classifiers to investigate the impacts of language tasks, recording media, and modalities on dementia assessment.

**Results:**

Our results show that (1) the ML classifiers trained with the picture description language task perform better than the classifiers trained with the story recall language task; (2) the data obtained from phone-based recordings improves the performance of ML classifiers compared to data obtained from web-based recordings; and (3) the ML classifiers trained with acoustic features perform better than the classifiers trained with linguistic features.

**Conclusion:**

This research demonstrates that we can improve the performance of automatic SLAMs as dementia assessment methods if we: (1) Use the picture description task to obtain participants’ speech; (2) Collect participants’ voices via phone-based recordings; and (3) Train ML classifiers using only acoustic features. Our proposed methodology will help future researchers to investigate the impacts of different factors on the performance of ML classifiers for assessing dementia.

## Introduction

More than 50 million people worldwide are living with different types of dementia, including *Alzheimer’s Disease* (AD) [1]. These are among the highest global diseases and have notable economic impacts on individuals and societies [2]. To mitigate the effects of dementia on older adults’ quality of life and help them plan for the future [3], detection of dementia as early as possible is necessary.

Identifying patients with dementia at the earliest stage of the disease can help them seek out different intervention programs [4] that could slow down disease progression and reduce its effect on their quality of life  [5]. Thus, clinicians have to run different *clinical assessment methods* (CAMs) (the abbreviations used in this paper have been listed in Table 1) such as *montreal cognitive assessment* (MoCA), which consists of a 30-points scales [6], to detect cognitive impairment associated with dementia [7] or to identify subjects with AD and *mild cognitive impairment* (MCI)^1^ [9].

**Table 1:** Lists of abbreviations

Abbreviation	Description
AI	Artificial intelligence
AD	Alzheimer’s disease
ANOVA	Analysis of variance
BI	Brunet’s index
CAMs	Clinical assessment methods
CNN	Convolutional neural network
COVFEFE	COre variable feature extraction feature
DB	Dementia bank
DL	Deep learning
DT	Decision trees
FK	Flesch–Kincaid
FRES	Flesch reading-ease
ET	Extra trees
HS	Honor’s statistic
LDA	Latent Dirichlet allocation
LSPs	Line spectral pairs
kNN	k-Nearest neighbor
Kurt	Kurtosis
MCI	Mild cognitive impairment
MFC	Mel frequency cepstrum
MFCCs	Mel frequency cepstral coefficients
MoCA	Montreal cognitive assessment
ML	Machine learning
mRMR	Minimal redundancy maximal relevance
NLTK	Natural language toolkit
PCA	Principle components analysis
POS	Part-of-speech
PDT	Picture description task
SA	Simple average
SIF	Smooth inverse frequency
SLAMs	Speech and language assessment methods
SRT	Story recall task
SVM	Support vector machine
std	Standard deviation
skew	Skewness
tf-IDF	Term frequency-inverse document frequency
WHO	World Health Organization
VP	Voicing probability

In the era of *artificial intelligence* (AI), clinicians can take advantage of AI-based assessment methods to detect dementia quickly. A good example of such methods is automatic *speech and language assessment method* (SLAM), which [10, 11] can detect speech and language impairments (e.g., difficulties with finding a relative expression, naming, and word comprehension and various level of language impairments [12]). These impairments are signs of the first cognitive manifestations of any types of dementia, specifically the onset of AD [12].

In this paper, we propose a methodology that can be followed to develop an automatic SLAM. Many studies  [13–17] proposed different approaches to develop automatic SLAMs. However, like most of the previous studies, we have not only focused on developing accurate classifiers to distinguish patients from healthy subjects, rather we have conducted different experiments to understand the impact of the language tasks, recording media, and types of features on the performance of *machine learning* (ML) classifiers. More specifically, we seek to find out the effect of (1) different language tasks, e.g., the *picture description task* (PDT) and the *story recall task* (SRT), (2) recording media, e.g., phone versus web-based interfaces, and (3) linguistic and acoustic features on the accuracy of our proposed methodology.

The contributions of this paper are threefold. First, we have compared the performance of ML classifiers trained with two different datasets obtained from different language tasks, PDT and SRT, and evaluated their performance in the classification task (i.e., classifying participants into patients with dementia and health controls). Our results show that the ML classifiers trained with data samples from the PDT perform better than the data samples obtained from SRT. Previous research rarely did such a comparison. Second, we have provided a comparison between voices recorded by phone and web-based recording media, and we have shown that the data obtained from phone-based recordings could improve the performance of ML classifiers compared to data obtained from web-based recordings. The prior research hardly considered the impact of recording channels on the quality of data that directly affects on the performance of ML classifiers. Finally, we have shown that the ML classifiers trained with acoustic features perform better than ML classifiers trained with linguistic features. Previous studies with other datasets showed that linguistic features perform better than acoustic features [18]. Unlike our dataset, datasets used by other studies were relatively old and used outdated technologies (e.g., tape recorders) to record human speeches which were noisy. Our dataset is very recent and the recording quality is better than the others which may be one of the reasons for getting more accurate results.

## Related works

Recent studies [19–25] showed how AI assessment systems and automatic SLAMs for dementia could be developed. SLAMs could help clinicians to: (1) Diagnose AD using patients’ spontaneous speech [26, 27]; (2) Detect cognitive decline using patients’ speech [28, 29]; (3) Distinguish patients with predementia from those with AD using patients’ speech [30]; (4) Identify AD using patients’ speech [18, 31]; (5) Develop AD risk assessment using patients’ speech [32]; (6) Detect AD using patients’ spontaneous speech [33] or using patients’ speech and transcriptions [34]; (7) Detect MCI using patients’ spontaneous speech [35]; (8) Distinguish patients with mild AD from those with MCI [36] and patients with AD from healthy controls using patients’ speech and transcriptions [20]; and (9) Diagnose AD using patients’ speech transcripts [15].

Most of the above-mentioned SLAMs [14, 15, 20, 27, 30, 37] have developed based on ML and *deep learning* (DL) algorithms [18, 26, 38] that have been trained using extracted linguistic and acoustic features from benchmark speech and language datasets. Examples of such datasets are *DementiaBank* (DB) dataset (i.e. the DB dataset is a collection of patients’ speech and transcriptions) [39];^2^ Pitt corpus^3^ [28, 40–43]; and * Alzheimer’s Dementia Recognition through Spontaneous Speech or ADReSS Challenge Dataset* [28] that has been developed by modifying the Pitt corpus. For example, in [44] a SLAM has been developed using lexical features which have been extracted from the DB. The SLAM which has been developed in [45] was trained by extracting linguistic and phonetic features from the Pitt corpus. The authors of [26, 26, 33] have developed SLAMs using the ADReSS dataset.

As mentioned earlier, developing SLAMs using ML [14, 30] and DL algorithms [18, 26, 38, 46–49] including *convolutional neural network* (CNN) [48]; Gated CNN [49] has been suggested by many studies. For example, in  [14, 30] *k-Nearest Neighbor* (kNN) was trained by these features to distinguish patients with MCI from healthy subjects with 63% accuracy. In  [13–15], two ML algorithms, *support vector machine* (SVM), *Decision Trees* (DT) were trained by features to detect voice impairments in patients with AD  [13] or predicting probable AD  [44] ( the SVM classifier was trained by linguistic features extracted from the DB dataset). In [50] a new ML algorithm named *emotional learning-inspired ensemble classifier* [51] was proposed to to develop an automatic SLAM.

The above mentioned studies have proved that automatic SLAMs can be used to assist clinicians to detect speech and language impairments associated with dementia in older adults. Therefore, it is worth investigating the methodologies that can be used to develop a SLAM considering the impacts of language tasks, recording media, and modalities.

## Methodology

Our methodology follows the following steps: (1) Collecting language datasets; (2) Using feature engineering methods: (2.1) Feature extraction to extract linguistic and acoustic features, (2.2) Feature selection to select informative features; (3) Training and evaluating different ML classifiers to investigate the impacts of language tasks, recording media and modalities on dementia assessment.

### Collecting language datasets

We have extracted patients’ speech and transcripts with various types of dementia^4^ as well as healthy control from a database named Talk2Me.^5^ The Talk2Me database contains speech data^6^ recorded using web or phone interfaces from participants while doing a variety of language tasks such as the PDT and SRT.^7^ The speech data of the PDT task,^8^ which aims to evaluate the semantic knowledge in subjects  [52], were collected from 3 participants without dementia and 5 participants with dementia.

The speech data of the SRT task,^9^ which has been used to assess impairment in episodic and semantic memory and also global cognition [52], were obtained from 10 participants without dementia and 4 participants with dementia.

Unlike other datasets reported in the literature, our dataset contains human speech recorded with phone-based and web-based interfaces. Therefore, although this dataset includes a limited number of data, we used it in our research because we are investigating the impact of recording interfaces on the performance of ML classifiers to assess dementia.

### Feature extraction

This section describes the linguistic and acoustic features that have discriminating characteristics to distinguish between healthy adults and people living with dementia.

#### Linguistic features

Using the *Natural Language Toolkit* [53] and other python libraries, we have extracted different types of linguistic features from the textual datasets that have been described in Table 2 (it lists the average number of sentences and words per individuals for different category of textual data sets). The linguistic features extracted for this study can be divided into three categories: (1) Lexical Features (e.g., lexical richness); (2) Syntactic Features [e.g., *part-of-speech* (POS)]; and (3) Semantic Features or Semantic-based Features.

**Table 2:** Statistics about our textual datasets

DATA	Average sentence	Standard deviation (sentence)	Average word	Standard deviation (word)
The PD task	9.0	4.4	153.5	97.92
The SR task	6.79	4.00	57.07	26.91
Recording media (Phone)	3.5	4.66	74.0	44.90
Recording media (Web)	2.27	1.25	65.59	31.11

*Lexical features* As lexical features, we have extracted features such as *Brunets Index* (BI) (see Eq. 1) and *Honors Statistic* (HS) with Eq. 2 [54]. These features have been proposed to measure the lexical richness. In Eqs. 1 and 2, *w* and *u* are the total number of word tokens and the total number of unique word types, respectively. We have also considered readability scores such as the *Flesch–Kincaid* (*FK*) (see Eq. 3), the *Flesch Reading-Ease* (FRES) Test (see Eq. 4) [55], to test the readability of the transcripts. Here, *s* and *SYL* indicate the total number of sentences and the total number of syllables, respectively.

1 $$BI= w^{(u^{-0.165})}$$

2 $$HS= \frac{100\log w}{1-\frac{w}{u}}$$

3 $$FK= 0.39 \left( \frac{w}{s} \right) + 11.8 \left( \frac{SYL}{w} \right) - 15.59$$

4 $$FRES= 206.835 - 1.015 \left( \frac{w}{s} \right) - 84.6 \left( \frac{SYL}{w} \right)$$

*Syntactic features* We have also extracted syntactic features such as POS ratios: (1) Third pronouns (3rd-pron-pers) to proper nouns (prop); (2) First pronouns (1st-pron-pers) to pronouns (1st-pron-pers);^10^ (3) Nouns to verbs; and (4) Subordinate to coordinate  [57] to calculate syntactical error in speech, which is indicative of *frontotemporal dementia* [58], and propositional and content density Eqs. 5 and 6 to quantify the syntax complexity. Here, *NN*, *VB*, *JJ*, *RB*, *IN*, and *CC* are the number of nouns, verbs, adjectives, adverbs, prepositions, and conjunctions respectively.

5 $$density_{p}= \frac{VB+JJ+RB+IN+CC}{N}$$

6 $$density_{c}= \frac{NN+VB+JJ+RB}{N}$$

*Semantic features* We suggest extracting semantic-based features that quantify speech incoherence and measure tangential speech.

To quantify speech incoherence, we calculated the similarity (Eq. 7) between sentence embeddings: ${v}_{s_{j}}$ using three sentence embeddings: *Simple Average* (SA)^11^ (see Eq. 8), or *Smooth Inverse Frequency* (SIF) embeddings^12^ [59] (see Eq. 10) and *term frequency-Inverse Document Frequency* (tf-IDF) (see Eq. 11). We proposed Eq. 12 to measure tangential speech or tangentiality in patients' speech with dementia [60], here, $N_{topic}$ is the optimal number of topics for a corpus made of interviews of subjects) employing *Latent Dirichlet Allocation* [61, 62].

7 $$Similarity_{{\textbf {SA}}} ({v}_{s_{i}},{v}_{s_{j}})= {{v}_{s_{i}} \cdot {v}_{s_{j}} \over \Vert {v}_{s_{i}}\Vert \Vert {v}_{s_{j}}\Vert }$$

8 $$Similarity_{{\textbf {SIF}}} ({v}_{s_{i}},{v}_{s_{j}})= 1-{{v}_{s_{i}} \cdot {v}_{s_{j}} \over \Vert {v}_{s_{i}}\Vert \Vert {v}_{s_{j}}\Vert }$$

9 $$Incoherence_{{\textbf {SA}}}=\min _{i}{\max _{j}{ Similarity_{{\textbf {SA}}} ({v}_{s_{i}},{v}_{s_{j}})\over {abs(i-j)+1} }}$$

10 $$Incoherence_{{\textbf {SIF}}}= \min _{i}{\sum _{j}{Similarity_{{\textbf {SIF}}} ({v}_{s_{i}},{v}_{s_{j}})\over {abs(i-j)+1} }}$$

11 $$Incoherence_{{\textbf {tf-IDF}}}= \min _{i}{\sum _{j}{Similarity_{{\textbf {TFIDF}}} ({v}_{s_{i}},{v}_{s_{j}})\over {abs(i-j)+1} }}$$

12 $$Tangentiality=1-\frac{N_{topic}}{\sum _{j}{N_{topic}}}$$

#### Acoustic features

We have extracted the acoustic features using the *COre Variable Feature Extraction Feature Extractor* (COVFEFE) tool [57]. Table 3 shows the list of features that we have considered in the study. The extracted features can be categorized into 3 groups: (1) Spectral Features; (2) Phonation and Voice Quality Features; and (3) Speech Features. We have considered the mean, *standard deviation* (std), *skewness* (skew) (lack of symmetry of a data distribution) and *kurtosis* (kurt) (measure of peakedness around the mean of a data distribution) of each acoustic features and also included the deltas of these features. In total, we extracted 296 features, but we only describe the features that are identified as meaningful by our feature selection methods.

**Table 3:** List of acoustic features that are considered in this research

Type	Name	Functional	# of Features
Spectral features	MFCCs 0–14	Mean, kurt, skew, std	60
	ΔMFCCs 0–14	Mean, kurt, skew, std	60
	log Mel freq 0–7	Mean, kurt, skew, std	32
	Δlog Mel freq 0–7	Mean, kurt, skew, std	32
	LSP freq 0–7	Mean, kurt, skew, std	32
	Δ LSP freq 0–7	Mean, kurt, skew, std	32
Phonation and voice	F0	Mean, kurt, skew, std	4
Quality features	ΔF0	Mean, kurt, skew, std	4
	Jitter local	Mean, kurt, skew, std	4
	Δjitter local	Mean, kurt, skew, std	4
	Jitter DDP	Mean, kurt, skew, std	4
	Δjitter DDP	Mean, kurt, skew, std	4
	Shimmer	Mean, kurt, skew, std	4
	Δshimmer	Mean, kurt, skew, std	4
	Loudness	mean, kurt, skew, std	4
	Δloudness	Mean, kurt, skew, std	4
Speech features	Voicing prob.	Mean, kurt, skew, std	4
	Δvoicing prob.	Mean, kurt, skew, std	4
Total			296

*Spectral features:* Spectral features encompasses the features derived from the *mel frequency cepstrum* (MFC) (i.e., MFC uses the Mel scale to represent short-term power spectrum of a sound), the *line spectral pairs* (LSPs) and *mel frequency cepstral coefficients* (MFCCs) ( it represent energy variations between frequency bands of a speech signal). Using MFCCs, we aim at accurately representing the phonemes articulated by speech organs (tongue, lips, jaws, etc.). Delta MFCCs are the trajectories of the MFCCs over time. The logarithm of Mel filter banks are calculated as an intermediate step of computing MFCCs and we have considered the *Log Mel Frequency Bands* and the *Delta Log Mel Frequency Bands* as spectral features. Previous research identified the MFCCs as one of the most relevant acoustic features to distinguish patients with different types of dementia [45, 63, 64]. Our analysis also confirm this claim (see Tables 4 and 7).

**Table 4:** Common acoustic features obtained by applying ANOVA, RF and mRMR feature selection methods on the recorded audio files of PDT and SRT

PD Task	SR task
MFCC 13 (mean)	MFCC 12,13 (skew)
MFCC 12,13 (kurt)	ΔMFCC 3,4,6,13 (mean)
MFCC 10,13 (skew)	ΔMFCC 4 (skew)
ΔMFCC 2,11 (mean)	ΔLSP freq 7 (mean)
ΔMFCC 2,3,6,7 (kurt)	ΔLSP freq 3,6 (kurt)
ΔMFCC 6,11,13 (skew)	Loudness (kurt, skew)
ΔLSP freq 3,5 (mean)	ΔLoudness (kurt, skew)
ΔLSP freq 2,6 (kurt)	F0 (kurt)
ΔLSP freq 1 (skew)	ΔF0 (mean)

LSPs are strongly related to underlying speech features and are thus useful in speech coding [65]. They are correlated to unvoiced speech, pause and silence which are reportedly effective in identifying linguistic impairments [66]. The delta of LSPs represents the change of LSPs over time. Our feature selection methods confirm the importance of LSPs and their deltas (see Tables 4 and 7).

*Phonation and voice quality features* This feature group includes *fundamental frequency* (F0), *Shimmer*, *Jitter*, *Loudness*, and the deltas of these features. The F0 feature is defined as the rate of oscillation of the vocal folds [67]. F0 is nearly periodic in speech of the healthy people but less periodic in patients [68]. Jitter describes frequency instability and shimmer is a measure of amplitude fluctuations. Loudness affects the amplitude of vibrations and it is correlated to the emotional states of the speaker  [69]. Previous studies reported that phonation and voice quality features are correlated with MCI and AD [70, 71], and our findings also support these claims (see Tables 4 and 7).

*Speech features* We have considered the *voicing probability* (VP) and the delta of voicing probability as relevant acoustic features. A voicing probability shows the percentage of unvoiced and voiced energy in a speech signal. A delta voicing probability indicates the rate of change over time. Our feature selection methods identified that mean, std and kurt of both features are discriminative features to identify older adults living with dementia (see Table 7).

### Feature selection and machine learning classifiers

To select the most informative set of features we used techniques such as *Principle Components Analysis* (PCA), *Analysis of Variance* (ANOVA), RF and  *Minimal Redundancy Maximal Relevance* (mRMR).

We trained different ML classifiers such as DT, *Extra Tree* (ET), kNN, SVM using a set of extracted linguistic and acoustic features, which have been already described.

## Results

This section investigates the performance of different ML algorithms trained using various features extracted from participants’ speeches during the PDT and SRT and these speeches have been collected using phone-based and web-based interfaces. We aim to show the impacts of the two language tasks: the PDT and SRT on the performance of ML classifiers.

Note that, we have trained the classifiers separately with linguistic and acoustic features, and therefore, in the following parts, we compare the performance of the classifiers developed with these two groups of features.

### Language tasks

This subsection investigates the impacts of the two language tasks: the PDT and SRT on the performance of ML classifiers.

#### PDT

**Fig. 1:**
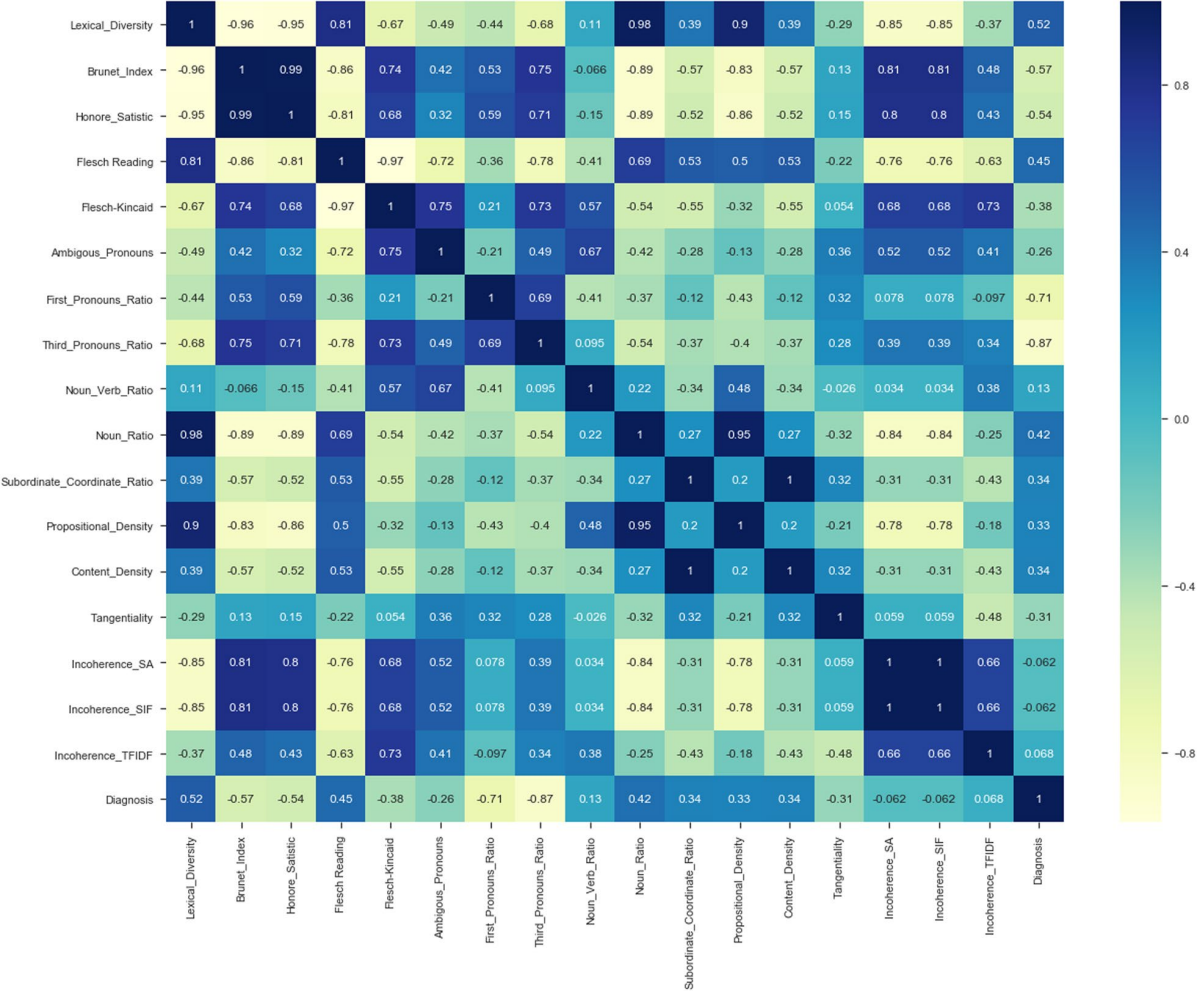
Correlation heat-map between 17 linguistic features extracted from textual data related to the PDT

We study the efficacy of linguistic (see Fig. 1) and acoustic features (see Table 4), which have been extracted from the speech of the participants without dementia ($N=3$) and participants with dementia ($N=5$) during the completion of PD task.

**Fig. 2:**
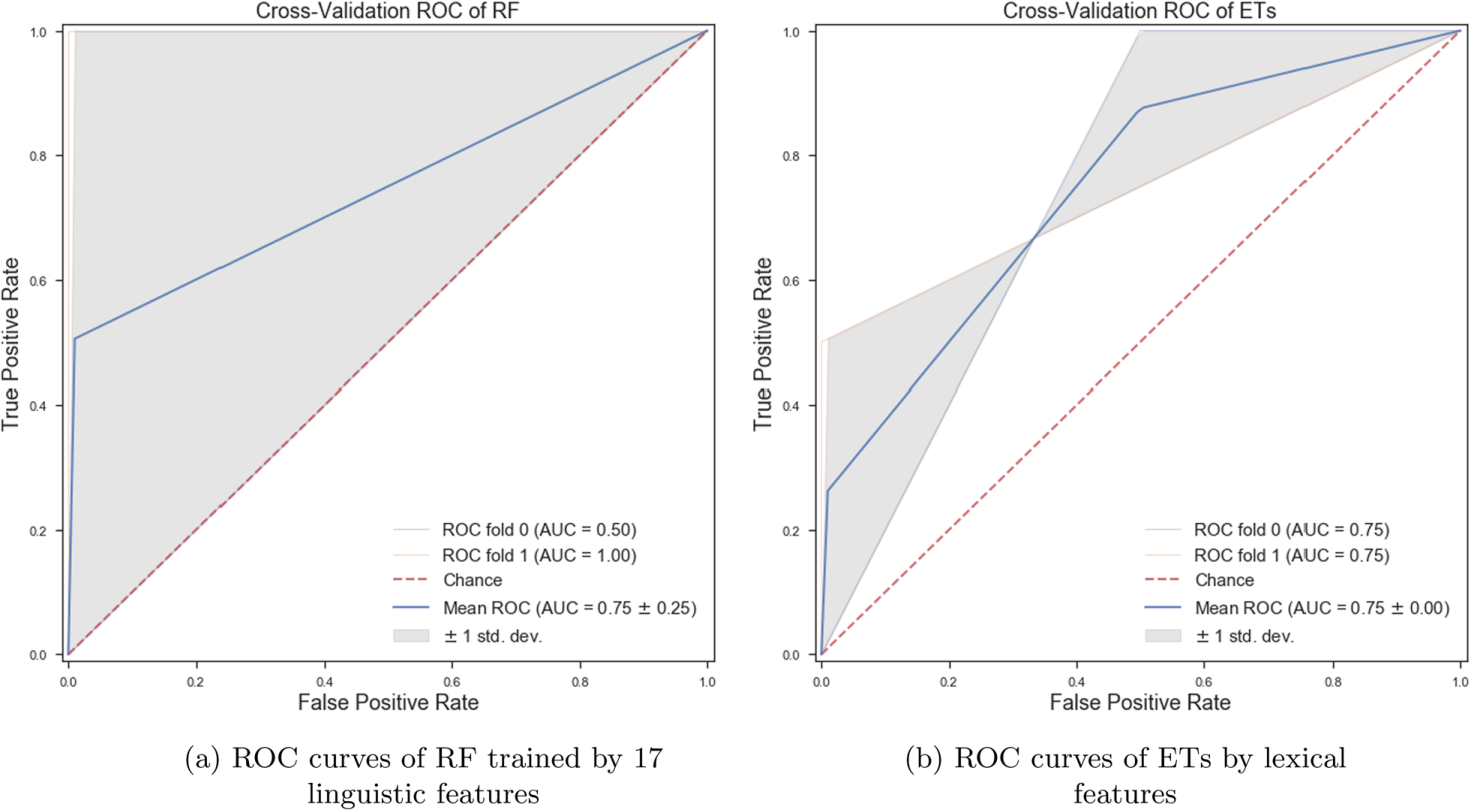
ROC curves of RF and ETs trained by different sets of linguistic features

**Fig. 3:**
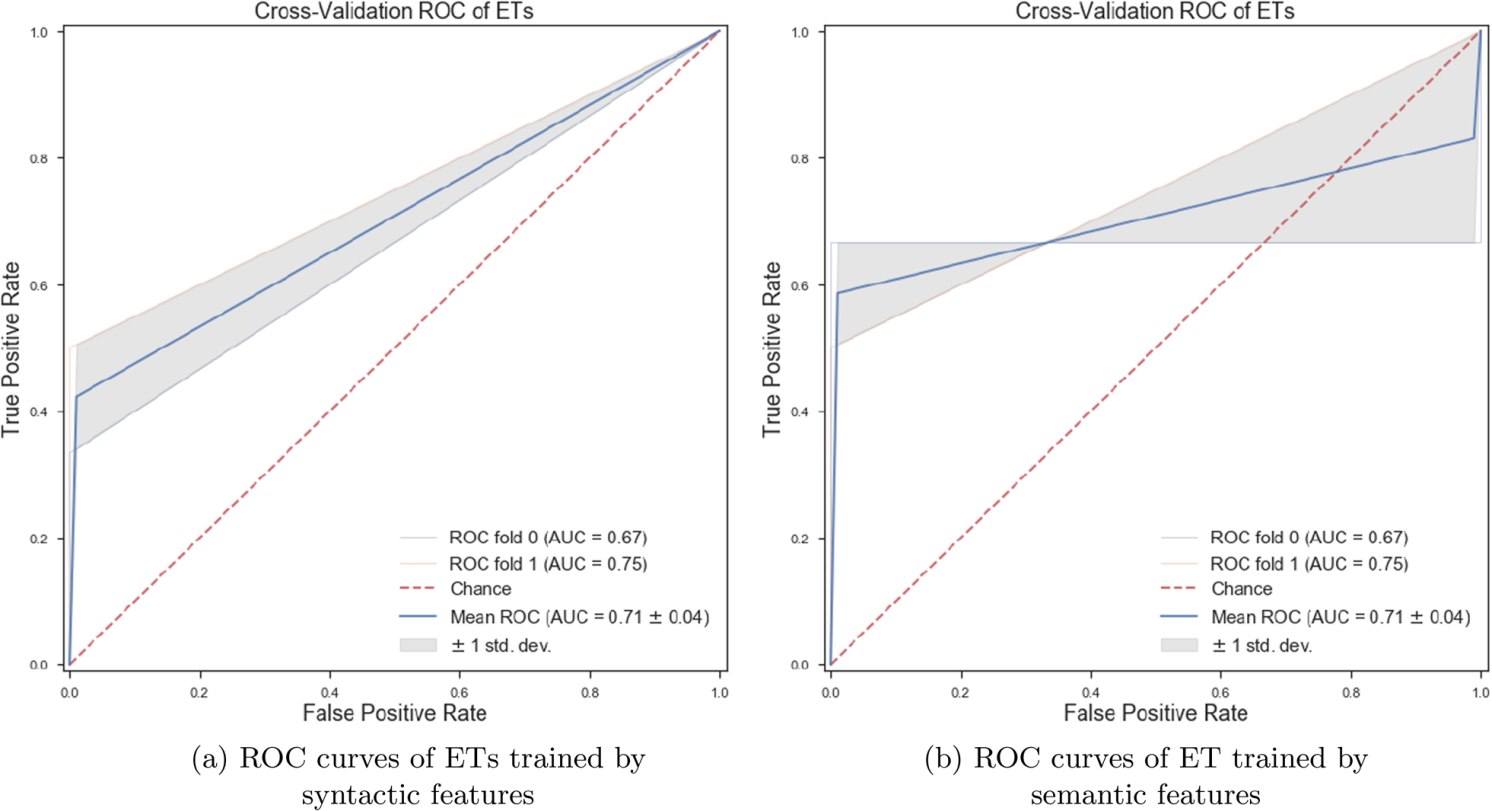
ROC curves of ETs trained by syntactic and semantic features

*Classifiers trained with linguistic features* We trained various ML classifiers using lexical, semantic, and syntactic features. Our obtained results show that if we train the ET algorithm with a set of lexical features, we can achieve more accurate classification results than other proposed ML algorithms in this paper (see Fig. 2b). Training ML algorithms with the set of lexical, semantic, and syntactic features could decrease the accuracy of classifiers (see Fig. 2a). By training the ML classifiers using 8 syntactic features, we observed the ET algorithm could classify patients from healthy controls with an accuracy of 63.0% (± 7%). By training various ML classifiers using 4 semantic features, we observed ET provide more accurate results than others and could classify the classes with an accuracy of 63.0% (± 7%) (see Fig. 3a, b).

**Fig. 4:**
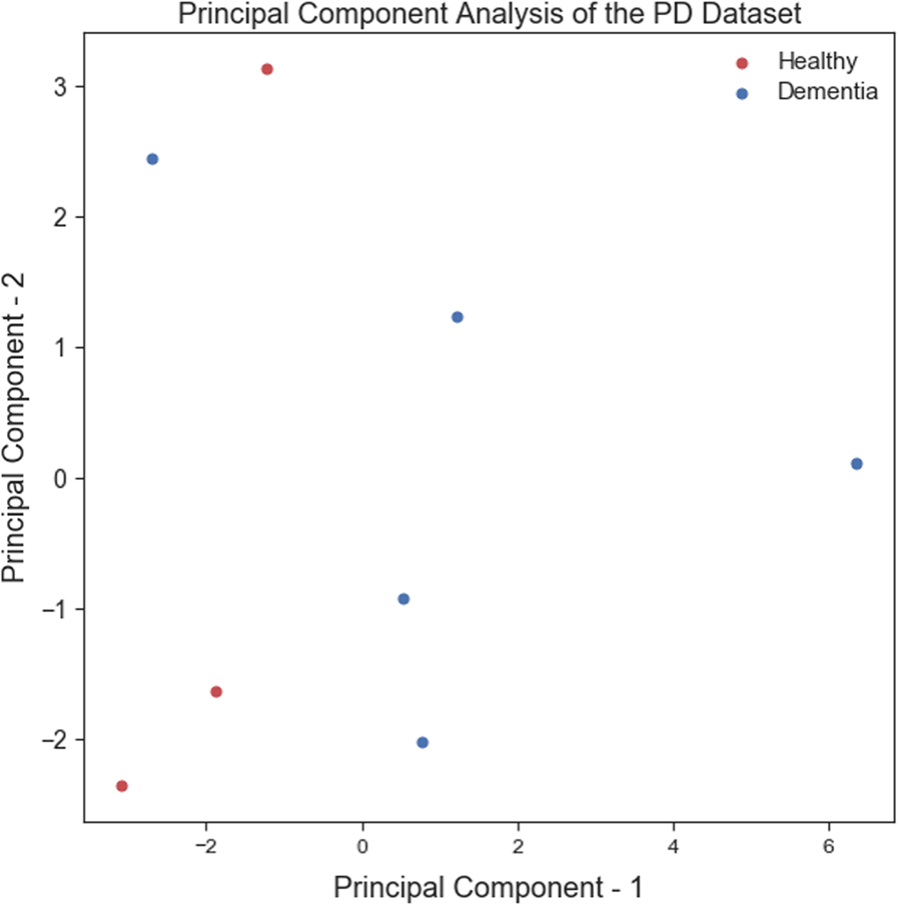
Using 2 PCs to separate subjects with dementia and healthy controls

**Fig. 5:**
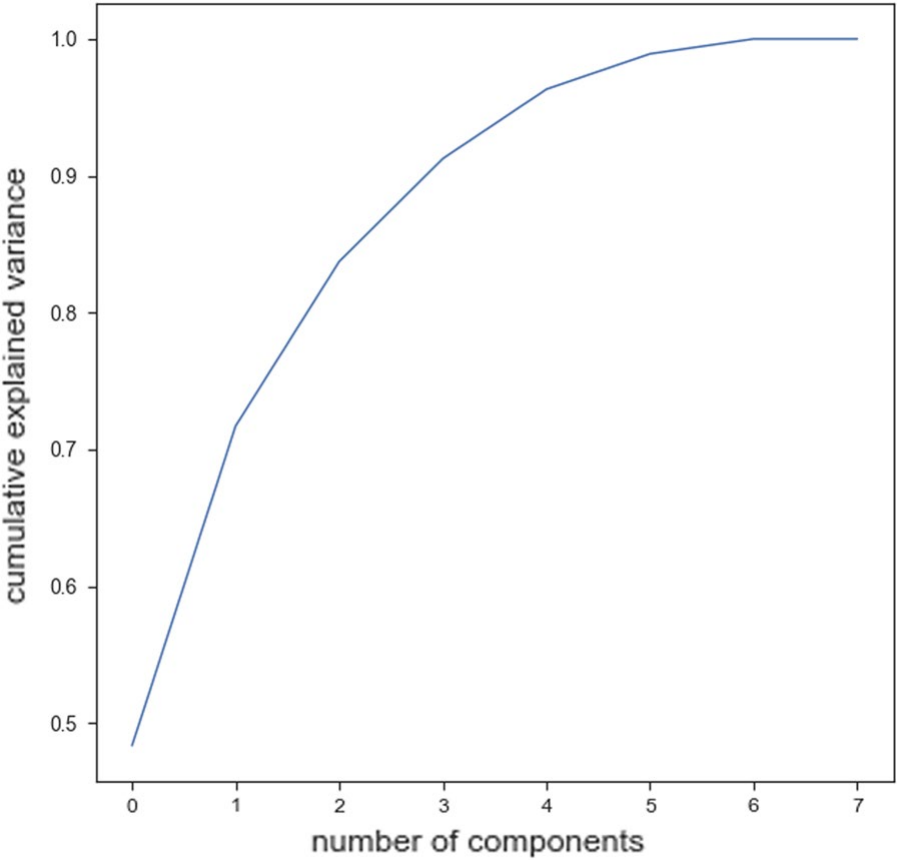
It presents the values of cumulative explained variance for different number of principle components

**Fig. 6:**
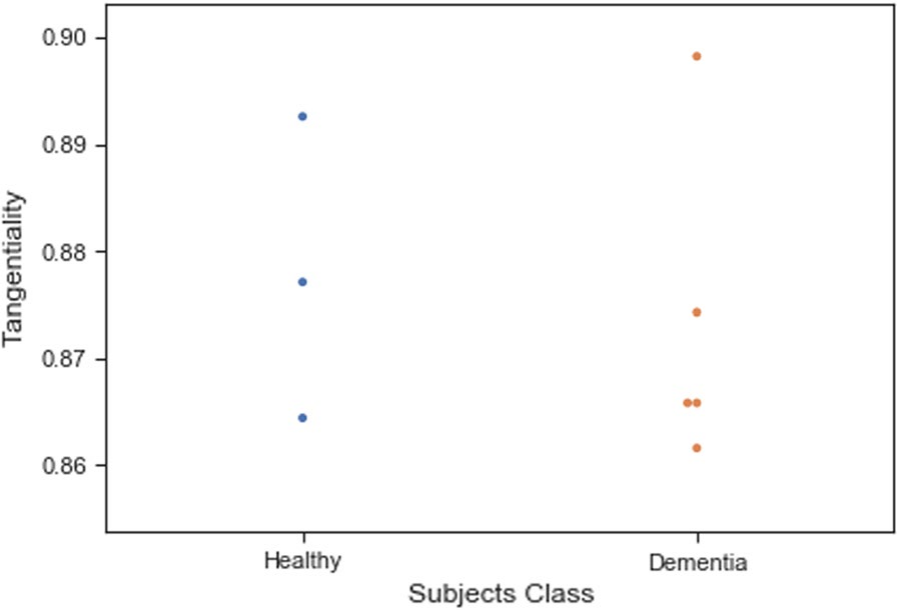
A comparison between the tangentiality measure for subjects with and without dementia

Training ML algorithms with 3 principle components (as Figs. 4 and 5 show using two principle components, health controls and patients might not be separated correctly) extracted from 17 features, we observed that the SVM algorithm with the linear kernel could classify with 63.0% (±  7%) accuracy. Furthermore, among lexical features, two Flesch–Kincaid ( CV^13^ = 23.17%, *p* value = 0.25) and FRE (CV = 15.15%, *p* value = 0.35) can provide better discrimination between these two groups of subjects, while the number of the third pronouns (the effect size equals to 1.319) and the first pronouns (the effect size equals to 2.198) among subjects without dementia has higher value than subjects with dementia. Thus, these two syntactic features might be considered as markers to detect subjects with MCI. Another interesting result is that measuring tangentiality (see Fig. 6) (with the effect size of 0.020) in speech can provide a better understanding to determine subjects with dementia from healthy subjects.

*Classifiers trained with acoustic features* Table 6 presents the classification results obtained by training ML classifiers using the acoustic features including the spectral (e.g., MFCC, LSP), speech (e.g., voicing probability), phonation (e.g., F0) and voice quality (e.g., jitter, shimmer) features as described in Sect. “Acoustic features”. We rank all these features using ANOVA, RF and mRMR methods and use the top 8 features identified by each of these methods to train the ML classifiers. Table 4 shows the top common acoustic features ranked by the above mentioned feature selection methods. We found that scikit-learn’s [72] default configurations work fine for the considered ML classifiers. Therefore, we use the default configurations for all classifiers. The F1 micro scores in Table 6 are obtained using the 3-fold cross-validation method. Our results show that the tree-based classifiers, e.g., RF, ET, and DT outperform the others.

#### SRT

This section presents the results obtained by training different ML classifier using linguistic and acoustic features extracted from participants’ speech and transcriptions produced by subjects without dementia ($N=10$) and patients with dementia ($N=4$) during the SRT.

**Fig. 7:**
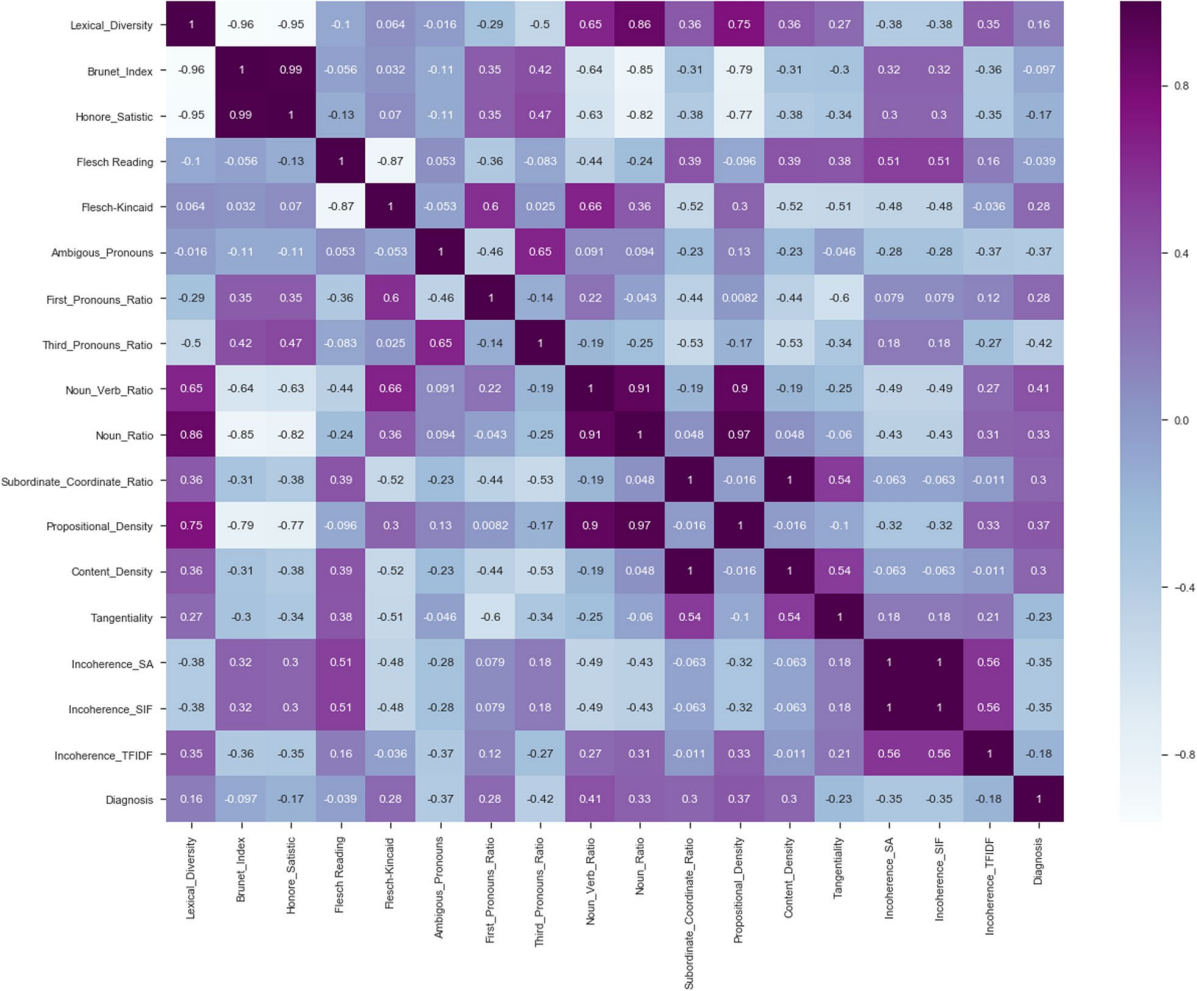
The correlation between different linguistic features extracted from the SRT

**Fig. 8:**
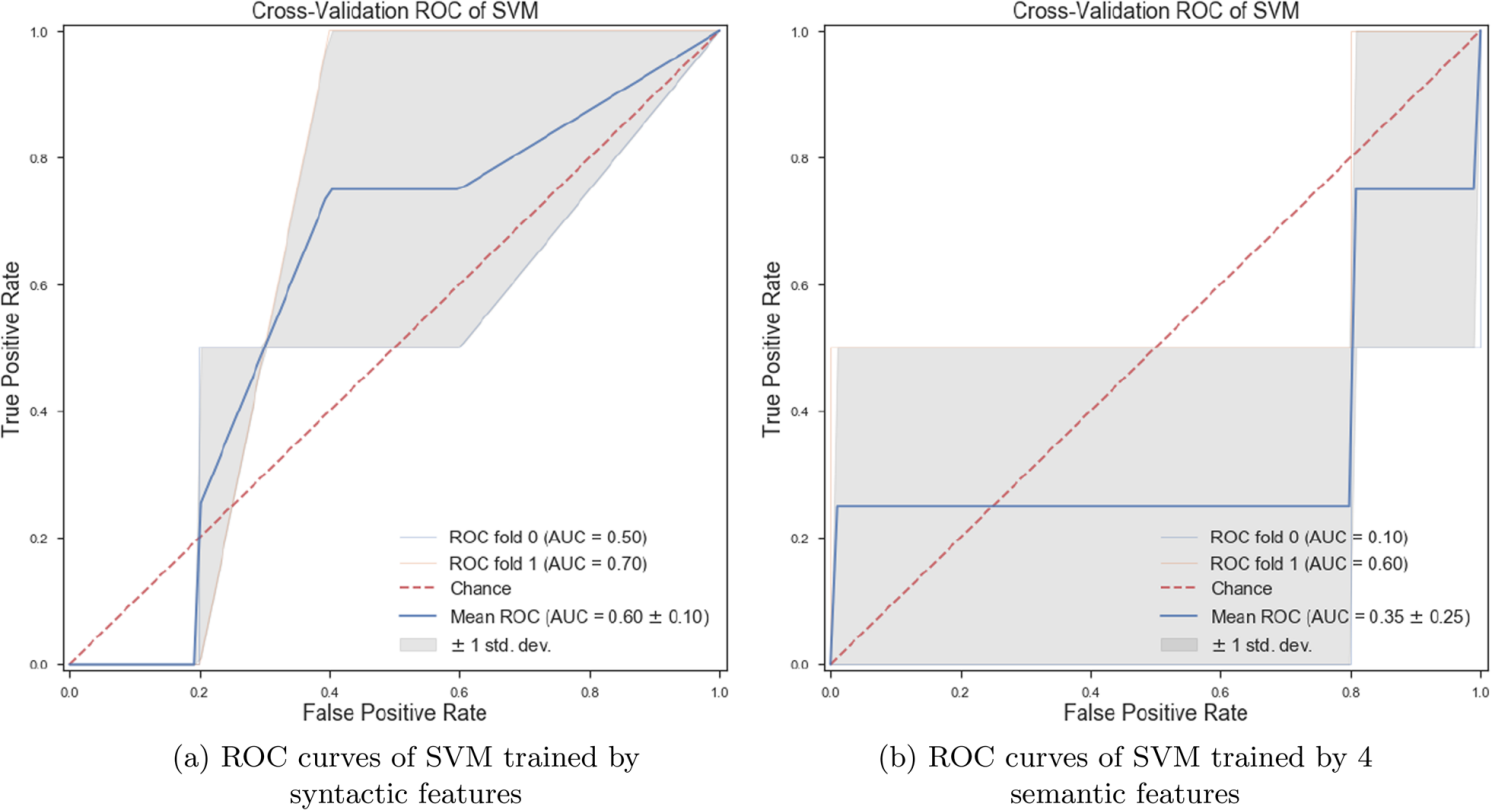
ROC curves of SVM trained with syntactic and semantic features

**Fig. 9:**
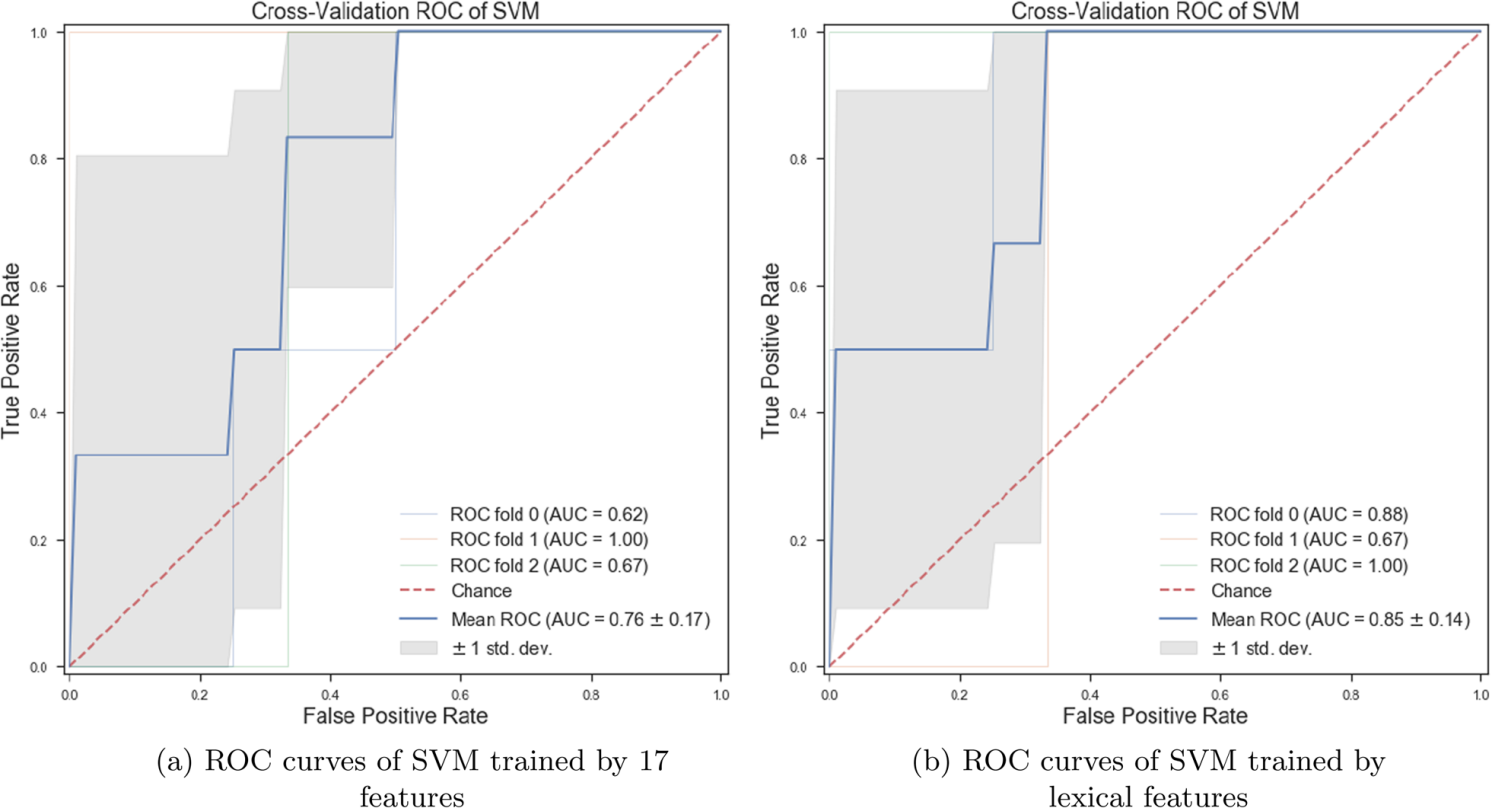
ROC curves of SVM trained by all and lexical features

**Fig. 10:**
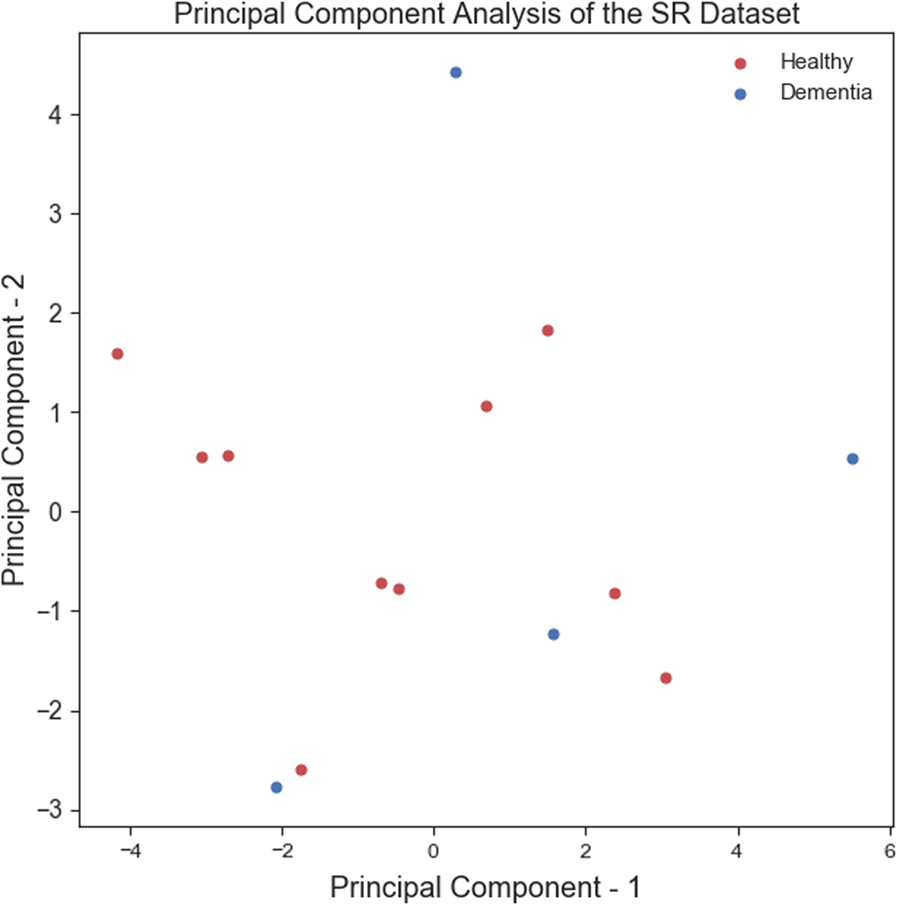
It shows that subjects with dementia and healthy controls cannot be linearly separated using 2 principle components

**Fig. 11:**
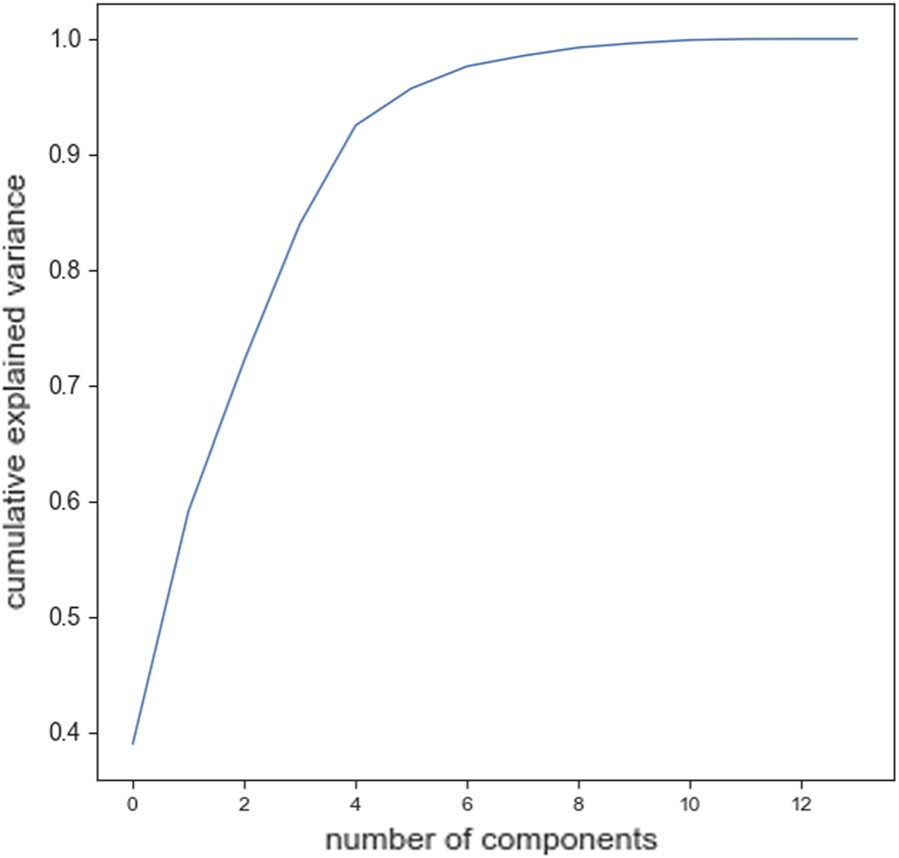
It presents the values of cumulative explained variance for different number of principle components

**Fig. 12:**
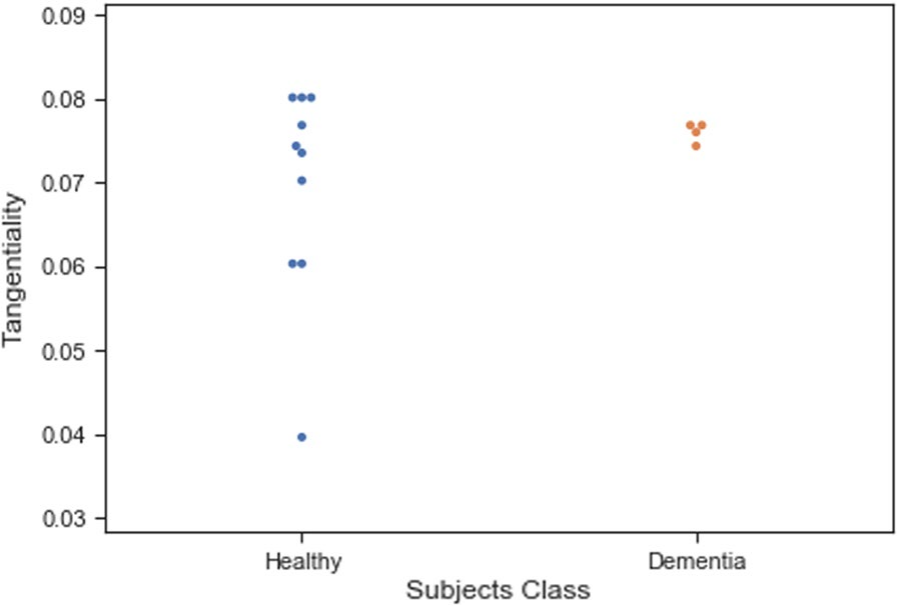
A comparison between the tangentiality measure for participants with dementia and healthy subjects

*Classifiers trained with linguistic features* We examined the performance of ML classifiers. trained using different linguistic features (see Fig. 7). Using 5 lexical features to train classifiers, the SVM (with the *Radial Basis Function* (RBF) kernel and $C=0.01$) and RF ($n\_estimators=2$ and $max\_depth=2$), could classify subjects with dementia and healthy subjects with 71% accuracy. We could get the same results using 8 syntactic features to train the SVM (with the RBF kernel and $C=0.01$) and RF($n\_estimators=2$ and $max\_depth=2$) (see Fig. 8a, b). Training the classifiers (3-fold Cross-Validation) with 17 lexical, semantic, and syntactic features, we concluded that the SVM (with the RBF kernel and $C=0.01$) could classify subjects with dementia and healthy adults with 72% accuracy (see Fig. 9a, b). Training ML algorithms with 3 *Principle Components* (PCs) (see Figs. 10 and 11) extracted from 17 features, we observed that the SVM algorithm with the RBF kernel could classify with 71% accuracy. Fig. 12 shows the comparison between the tangentiality measure for subjects with dementia and healthy subjects in speech provide during the SRT.

*Classifiers trained with acoustic features* We trained the ML algorithms using the top 15 acoustic features following the same methodology that we used in Sect. “PDT”. More specifically, we extracted various types of acoustic features from audio data collected from participants without dementia ($N=10$) and subjects with dementia ($N=4$). We ranked them (see Table 4 which shows the top common acoustic features) using ANOVA, RF, and mRMR feature selection methods. Note that we used scikit-learn’s default configurations for all classifiers of this sub-section. The F1 micro scores in Table 6 are obtained using the 3-fold cross-validation method.

#### Comparison between PDT and SRT

**Fig. 13:**
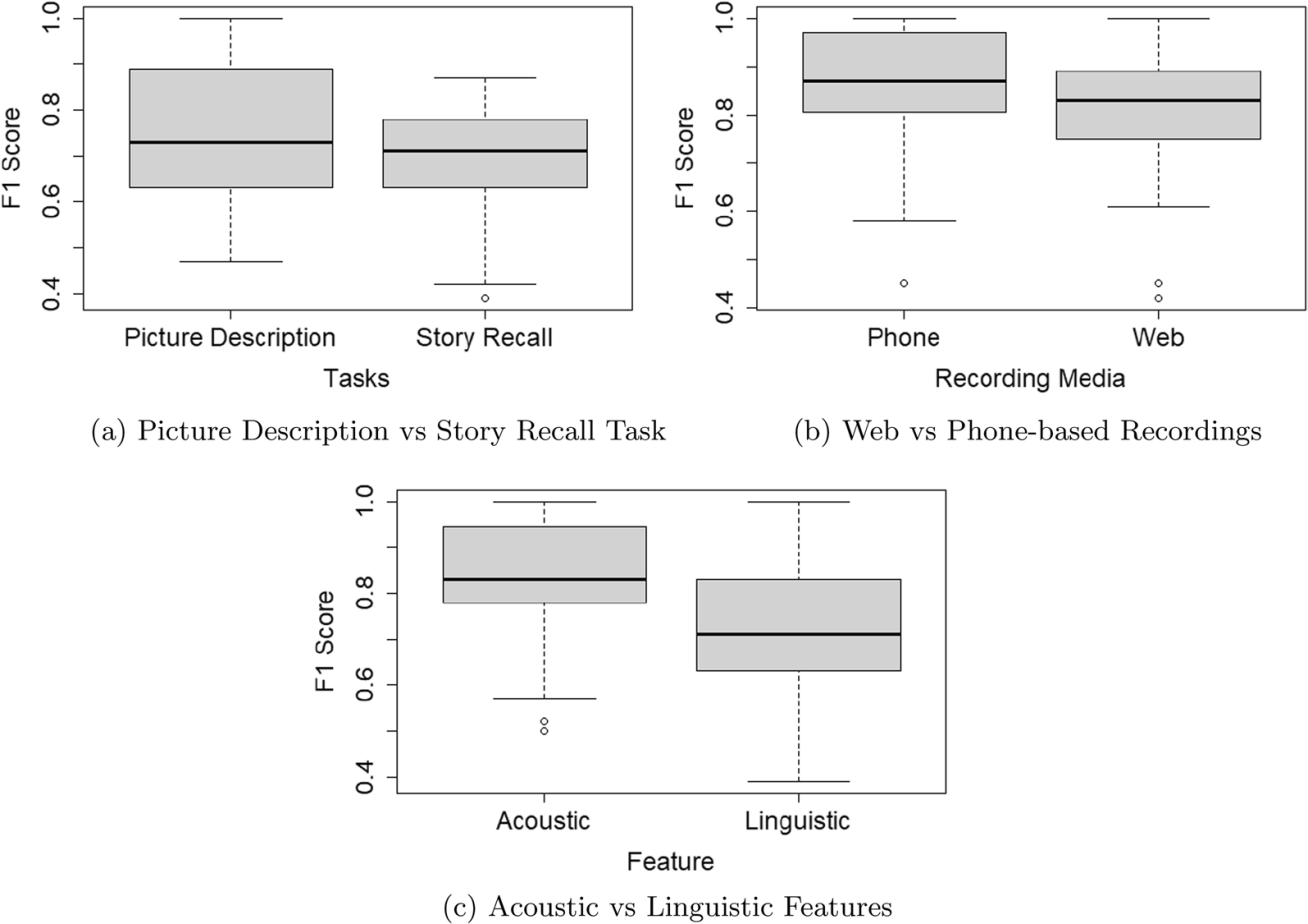
Boxplots showing the F1 scores obtained from different classifiers: **a** distributions of the F1 scores in the picture description and story recall tasks, **b** distributions of the F1 scores for web and phone-based recordings, and **c** distributions of the F1 scores in the linguistic and acoustic features. These boxplots show that the picture description task, phone-based recordings, and acoustic features provide better performance than the story recall task, web-based recordings, and linguistic features

As mentioned earlier, we have also evaluated the impact of language tasks on the performance of ML classifiers for detecting patients with dementia. We have used audio recordings and transcribed textual datasets to extract linguistic and acoustic features from speech and language datasets obtained from PDT and SRT. Our datasets are imbalanced and therefore micro F1 scores are more appropriate to report the performance of the ML classifiers. To assess the efficiency of PDT and SRT, we have calculated a range of F1 scores using different feature sets and classifiers as shown in Tables 5 and  6. We have used lexical, syntactic, semantic, and a combination of all these 3 feature groups as linguistic features. For acoustic features, we have used ANOVA, RF, and mRMR feature selection methods. We have also used the common features in these 3 feature selection methods as another set of acoustic features. Finally, we have applied DT, ET, Linear SVM, RBF SVM, *Linear Discriminant Analysis* (LDA), *Logistic Regression* (LR), kNN and RF algorithms to compute the F1 scores. Fig. 13a shows the distributions of F1 scores for PD and SR tasks. A one-way ANOVA test performed on the F1 scores of the PD and SR tasks shows that the means are significantly different (F(1,126) = 8.27, *p* = 0.005). A Tukey’s post-hoc test shows that the mean F1 scores of datasets from the PDT are higher than the SR task (*p* = 0.005), i.e., the ML classifiers trained by datasets obtained from using the PDT (i.e., the Cookie Theft picture) perform better than those classifiers trained by datasets obtained from the SRT.

**Table 5:** F1 (micro) scores obtained by applying ML algorithms on linguistic features

Features	Algorithms	PDT	SRT	Web	Phone
Lexical	DT	0.63 (± 0.07)	0.71 (± 0.00)	0.42 (± 0.17)	0.92 (± 0.17)
	ET	0.73 (± 0.13)	0.57 (± 0.57)	0.83 (± 0.00)	0.92 (± 0.17)
	kNN	0.52 (± 0.29)	0.42 (± 0.00)	0.45 (± 0.00)	0.45 (± 0.00)
	LDA	0.63 (± 0.07)	0.63 (± 0.07)	0.75 (± 0.17)	0.92 (± 0.17)
	R_SVM	0.63 (± 0.07)	0.71 (± 0.00)	0.83 (± 0.00)	0.83 (± 0.00)
	L_SVM	0.63 (± 0.07)	0.71 (± 0.00)	0.83 (± 0.00)	1.00 (± 0.00)
	LR	0.63 (± 0.07)	0.63 (± 0.07)	0.83 (± 0.00)	1.00 (± 0.00)
	RF	0.47 (± 0.27)	0.71 (± 0.00)	0.83 (± 0.00)	0.92 (± 0.17)
Syntactic	DT	0.73 (± 0.13)	0.57 (± 0.00)	0.83 (± 0.00)	0.83 (± 0.00)
	ET	0.80 (± 0.40)	0.64 (± 0.14)	0.83 (± 0.00)	0.83 (± 0.00)
	kNN	0.69 (± 0.63)	0.53 (± 0.23)	0.45 (± 0.00)	0.45 (± 0.00)
	LDA	0.37 (± 0.07)	0.50 (± 0.43)	0.75 (± 0.17)	0.75 (± 0.50)
	R_SVM	0.63 (± 0.07)	0.71 (± 0.00)	0.83 (± 0.00)	0.83 (± 0.00)
	L_SVM	0.63 (± 0.07)	0.71 (± 0.00)	0.83 (± 0.00)	0.67 (± 0.33)
	LR	0.80 (± 0.40)	0.71 (± 0.00)	0.83 (± 0.00)	0.75 (± 0.17)
	RF	0.47 (± 0.27)	0.57 (± 0.00)	0.75 (± 0.17)	0.92 (± 0.17)
Semantic	DT	0.53 (± 0.27)	0.64 (± 0.14)	0.83 (± 0.00)	0.83 (± 0.33)
	ET	0.57 (± 0.47)	0.71 (± 0.29)	0.83 (± 0.00)	0.83 (± 0.00)
	kNN	0.69 (± 0.63)	0.53 (± 0.23)	0.45 (± 0.00)	0.45 (± 0.00)
	LDA	0.63 (± 0.07)	0.71 (± 0.00)	0.83 (± 0.00)	0.58 (± 0.50)
	R_SVM	0.63 (± 0.07)	0.71 (± 0.00)	0.83 (± 0.00)	0.83 (± 0.00)
	L_SVM	0.63 (± 0.07)	0.71 (± 0.00)	0.83 (± 0.00)	0.83 (± 0.00)
	LR	0.63 (± 0.07)	0.50 (± 0.43)	0.83 (± 0.00)	0.83 (± 0.00)
	RF	0.73 (± 0.13)	0.57 (± 0.00)	0.83 (± 0.00)	0.83 (± 0.00)
All	DT	0.73 (± 0.13)	0.64 (± 0.14)	0.75 (± 0.17)	1.00 (± 0.00
	ET	0.63 (± 0.07)	0.79 (± 0.14)	0.83 (± 0.00)	0.75 (± 0.50)
	kNN	0.52 (± 0.29)	0.39 (± 0.05)	0.45 (± 0.00)	0.45 (± 0.00)
	LDA	0.63 (± 0.07)	0.64 (± 0.14)	0.75 (± 0.17)	0.75 (± 0.50
	R_SVM	0.63 (± 0.07)	0.71 (± 0.00)	0.83 (± 0.00)	0.83 (± 0.00)
	L_SVM	0.63 (± 0.07)	0.64 (± 0.14)	0.75 (± 0.17)	1.00 (± 0.00)
	LR	0.70 (± 0.60)	0.64 (± 0.14)	0.83 (± 0.00	0.75 (± 0.17)
	RF	0.63 (± 0.07)	0.71 (± 0.00)	0.83 (± 0.00)	1.00 (± 0.00)

**Table 6:** F1 (micro) scores obtained by training ML algorithms with acoustic features

Features	Algorithms	PDT	SRT	Web	Phone
ANOVA	DT	0.83 (± 0.24)	0.50 (± 0.24)	0.89 (± 0.16)	0.81 (± 0.02)
	ET	0.98 (± 0.03)	0.86 (± 0.09)	0.83 (± 0.24)	0.93 (± 0.09)
	kNN	0.83 (± 0.24)	0.78 (± 0.02)	0.89 (± 0.16)	0.93 (± 0.09)
	LDA	0.89 (± 0.16)	0.70 (± 0.14)	0.89 (± 0.16)	1.00 (± 0.00)
	R_SVM	0.72 (± 0.21)	0.78 (± 0.02)	0.89 (± 0.16)	0.76 (± 0.06)
	L_SVM	0.83 (± 0.24)	0.70 (± 0.14)	0.72 (± 0.21)	1.00 (± 0.00)
	LR	0.83 (± 0.24)	0.78 (± 0.02)	0.72 (± 0.21)	0.93 (± 0.09)
	RF	0.99 (± 0.02)	0.83 (± 0.06)	0.83 (± 0.24)	0.93 (± 0.09)
RF	DT	0.72 (± 0.21)	0.57 (± 0.17)	1.00 (± 0.00)	0.87 (± 0.09)
	ET	0.99 (± 0.02)	0.80 (± 0.04)	1.00 (± 0.00)	0.99 (± 0.02)
	kNN	0.89 (± 0.16)	0.78 (± 0.02)	0.89 (± 0.16)	0.93 (± 0.09)
	LDA	1.00 (± 0.00)	0.57 (± 0.17)	0.89 (± 0.16)	0.93 (± 0.09)
	R_SVM	0.61 (± 0.08)	0.78 (± 0.02)	0.61 (± 0.08)	0.76 (± 0.06)
	L_SVM	0.89 (± 0.16)	0.78 (± 0.02)	0.72 (± 0.21)	1.00 (± 0.00)
	LR	0.89 (± 0.16)	0.87 (± 0.09)	0.72 (± 0.21)	0.93 (± 0.09)
	RF	1.00 (± 0.00)	0.78 (± 0.02)	0.90 (± 0.14)	1.00 (± 0.00)
mRMR	DT	1.00 (± 0.00)	0.70 (± 0.14)	0.83 (± 0.24)	0.87 (± 0.09)
	ET	1.00 (± 0.00)	0.81 (± 0.05)	0.97 (± 0.05)	1.00 (± 0.00)
	kNN	0.50 (± 0.14)	0.78 (± 0.02)	1.00 (± 0.00)	0.81 (± 0.16)
	LDA	1.00 (± 0.00)	0.77 (± 0.21)	1.00 (± 0.00)	1.00 (± 0.00)
	R_SVM	0.61 (± 0.08)	0.78 (± 0.02)	0.72 (± 0.21)	0.76 (± 0.06)
	L_SVM	0.78 (± 0.31)	0.50 (± 0.08)	1.00 (± 0.00)	0.87 (± 0.19)
	LR	0.78 (± 0.31)	0.78 (± 0.02)	1.00 (± 0.00)	0.87 (± 0.19)
	RF	0.99 (± 0.02)	0.78 (± 0.02)	0.88 (± 0.16)	1.00 (± 0.00)
Common	DT	1.00 (± 0.00)	0.52 (± 0.37)	1.00 (± 0.00)	0.80 (± 0.28)
	ET	1.00 (± 0.00)	0.84 (± 0.11)	0.74 (± 0.21)	0.94 (± 0.08)
	kNN	0.83 (± 0.24)	0.70 (± 0.14)	0.89 (± 0.16)	1.00 (± 0.00)
	LDA	0.78 (± 0.16)	0.80 (± 0.16)	0.89 (± 0.16)	0.87 (± 0.19)
	R_SVM	0.72 (± 0.21)	0.78 (± 0.02)	0.78 (± 0.16)	0.76 (± 0.06)
	L_SVM	0.83 (± 0.24)	0.77 (± 0.21)	0.72 (± 0.21)	1.00 (± 0.00)
	LR	0.83 (± 0.24)	0.70 (± 0.14)	0.72 (± 0.21)	1.00 (± 0.00)
	RF	0.98 (± 0.02)	0.78 (± 0.02)	0.81 (± 0.20)	0.95 (± 0.07)

### Recording media

We were also interested in figuring out the direct effect of using the web interface or phone interface on the quality of recorded language data that indirectly impacts the performance of ML classifiers.

#### Classifiers trained with linguistic features

We have trained various ML classifiers using linguistic features extracted from recorded language data (10 samples related to subjects without dementia and 2 samples related to subjects with dementia) that were collected using the phone interface and web interface. Table 6 shows that the classification results obtained from the web-interface data are more accurate than the results obtained from the phone-interface data. Using 5 lexical features, the SVM (with the linear kernel) classifier and the LR can classify samples with 99.9% accuracy. However, using 8 syntactic features, we drop all ML classifiers’ performance, including the SVM (with the linear kernel); thus, the SVM can determine subjects with dementia with 83% accuracy. Similarly, if we use 4 semantic features including incoherence and tangential metric to train classifiers, they can provide better performance than using 8 syntactic features. Note that the datasets are imbalanced data, so the obtained accuracy might be changed by having more samples from patients with dementia.

#### Classifiers trained with acoustic features

We have developed the classifiers using the acoustic features extracted from the audio files. We used 16 phone-based recordings from 3 healthy adults and 1 dementia patient (each participant attended 4 sessions). Similarly, we have considered 8 web-based recordings from subjects with dementia ($N=3$) and subjects with dementia ($N=5$) (only 1 session each). We have followed the same methodology to rank the acoustic futures as we described before for the acoustic features. Table 7 shows the common features ranked by ANOVA, RF, and mRMR methods. We use the top 15 features to train the classifiers. Table 6 shows the F1 scores obtained from the DT, ET, Linear SVM, RBF SVM, LDA, LR, kNN and RF algorithms. We have used scikit-learn’s default configurations and the 3-fold cross-validation method to calculate the F1 scores. We found that DT perform better for web-based recordings and linear the SVM showed better performance for phone-based recordings. Note that the datasets are imbalanced data, so the obtained results here might be changed by having more samples from patients with dementia.

**Table 7:** Common acoustic features obtained by applying ANOVA, RF and mRMR feature selection methods on phone and web-based recordings

Web	Phone
MFCC 5, 11, 12 (mean)	MFCC 6, 9 (std)
ΔMFCC 11, 13 (mean)	MFCC 3 (skew)
ΔMFCC 0, 3, 6, 9, 10 (skew)	MFCC 3, 5 (kurt)
Δlog Mel freq 0, 5, 6 (skew)	ΔMFCC 0 (std)
Voicing prob. (kurt, std)	LSP freq 7 (mean)
ΔVoicing prob. (kurt, mean, std)	LSP freq 2, 3, 4 (skew)
LSP freq 0 (kurt)	LSP freq 1 (kurt)
F0 (skew)	ΔLSP freq 3 (mean)
Jitter local (kurt, skew)	ΔLSP freq 5 (skew)
ΔJitter local (kurt)	log Mel freq 2 (skew)
ΔJitter DDP (kurt)	Δlog Mel freq 1, 2, 3 (std)
ΔShimmer local (kurt)	Voicing prob. (kurt, std)
	Loudness (kurt)

#### Comparison between phone-based and web-based recordings

We have performed a one-way ANOVA test on the F1 scores of the phone and web-based recordings as shown in Tables  5 and  6. Our analysis shows that the means of these 2 groups are significantly different (F(1,126) = 4.26, *p* = 0.04). Figure 13b shows the distributions of F1 scores of these 2 groups. A Tukey’s post-hoc test shows that the mean F1 scores of the classifiers developed by the extracted features from the phone-based recordings are higher than web-based recordings (*p* = 0.04), i.e., the ML classifiers trained with the phone-based recordings perform better than the web-based recordings.

### Comparison between linguistic and acoustic features

Tables 5 and  6 show the results obtained by using different linguistic and acoustic features. We consider all F1 scores (total 256) to compare the performance between the classifiers trained with linguistic and acoustic features. Figure 13c shows the distributions of F1 scores of these 2 groups. A one-way ANOVA test performed on the F1 scores shows that the means are significantly different (F(1, 256) = 62.43, *p* $\approx$ 0). A Tukey’s test for post-hoc analysis shows that the mean F1 scores of the classifiers trained with the acoustic features are higher than the classifiers trained with the linguistic features (*p* = 0). That is, the ML classifiers trained with the acoustic features perform better than the classifiers trained with the linguistic features.

## Discussion

This research has focused on evaluating the impacts of language tasks, recording media, and modalities on the performance of ML classifiers that can be used for dementia assessment. This section discusses various aspects of our methodology including generalization, validity, reliability, and fairness.

### Generalization: selecting meaningful features

One of the problems we have faced with the acoustic features is that when we have applied ANOVA, RF, and mRMR feature selection methods on different datasets (i.e., obtained from various recording media or language tasks), each time we have received different sets of features (see Tables 4 and 7). Therefore, we are interested in combining all features so that we get almost consistent performance with all datasets. For this purpose, we have used PCA to combine a group of features, as shown in Table 8. We have considered MFCCs (0–14th order coefficients), the deltas of these MFCCs, and the deltas of LSP frequency bands (0–7) in the PCA because these groups of features appear more frequently in our rankings (see Tables 4 and 7). We found that the first two PCs can retain, on average 75% of the variance, and hence we have considered only the first 2 PCs to train the classifiers. Table 9 shows how these PCA features perform with 4 different sets of data. Our results show that we achieved almost consistent performance with the tree-based classifies ranging from 78 to 93% F1 scores with these generalized sets of features.

**Table 8:** Generalization—Combine the acoustic features using PCA

Feature Name	Functional	Principle Component (PC)
MFCC 0-14	Mean	1st PC from the means of 15 MFCCs
		2nd PC from the means of 15 MFCCs
	Kurt	1st PC from the kurt of 15 MFCCs
		2nd PC from the kurt of 15 MFCCs
	Skew	1st PC from the skew of 15 MFCCs
		2nd PC from the skew of 15 MFCCs
ΔMFCC 0-14	mean	1st PC from the means of 15 ΔMFCCs
		2nd PC from the means of 15 ΔMFCCs
	Kurt	1st PC from the kurt of 15 ΔMFCCs
		2nd PC from the kurt of 15 ΔMFCCs
	Skew	1st PC from the skew of 15 ΔMFCCs
		2nd PC from the skew of 15 ΔMFCCs
ΔLSP freq 0-7	mean	1st PC from the means of 8 ΔLSP freq
		2nd PC from the means of 8 ΔLSP freq
	Kurt	1st PC from the kurt of 8 ΔLSP freq
		2nd PC from the kurt of 8 ΔLSP freq
	Skew	1st PC from the skew of 8 ΔLSP freq
		2nd PC from the skew of 8 ΔLSP freq

**Table 9:** Results obtained by applying ML algorithms on PCA-based acoustic features that are extracted from all datasets

Classifier	PDT	SRT	Web	Phone
DT	0.78 (± 0.16)	0.78 (± 0.02)	0.72 (± 0.21)	0.80 (± 0.16)
ET	0.61 (± 0.28)	0.61 (± 0.28)	0.68 (± 0.22)	0.92 (± 0.10)
kNN	0.61 (± 0.08)	0.78 (± 0.02)	0.61 (± 0.08)	0.80 (± 0.28)
LDA	0.50 (± 0.14)	0.50 (± 0.14)	0.50 (± 0.14)	0.87 (± 0.09)
R_SVM	0.61 (± 0.08)	0.65 (± 0.18)	0.72 (± 0.21)	0.76 (± 0.17)
L_SVM	0.50 (± 0.14)	0.35 (± 0.33)	0.89 (± 0.16)	0.67 (± 0.25)
LR	0.72 (± 0.21)	0.22 (± 0.16)	0.89 (± 0.16)	0.60 (± 0.28)
RF	0.54 (± 0.15)	0.78 (± 0.02	0.79 (± 0.23)	0.93 (± 0.07)

### Validity and reliability

We can assess the validity and reliability of a classifier using the *Intra-class Correlation Coefficient* (ICC) [73] and the *Pearson Correlation Coefficient* (PCC) [73] (i.e., if PCC returns a value close to 1, then the classifier provides valid results; however, if the value is below, 0.5 indicates less correlation and validation). We observed that PCC value obtained from our classifiers trained with linguistic features is higher than 0.5.

### Fairness and explainability

In general, ML classifiers for developing automatic SLAMs are supervised classifiers, and therefore they are prone to producing unfair results. In our work, we tried not to consider sensitive attributes such as gender and race as features [74]. However, we are working on different verbal tasks that might slightly be influenced by gender differences [75]. Another issue is that this type of assessment tool compares the user’s language against similar users who are assumed to have AD or MCI [76]. Another essential attribute that might affect the fairness of automatic SLAMs is the level of education. It has been shown that some SLAMs cannot provide accurate diagnostics when there are subjects with lower education levels among the population of study [77]. SLAMs require a set of mechanisms to ensure that end-users trust in their performances and know how the system provides output. It is essential to motivate people to adopt not only the methods but also to share their data. Fairness is an essential concern, especially as automatic SLAMs are being deployed more broadly in detecting other types of mental health problems. Fairness, in the end, comes down to the robustness aspect. When we create SLAMs, we want them to be fair, and this means robust when deployed in different geographic settings and populations. Automatic SLAMs should be accurate and explainable to be adopted by psychiatrists during their assessment procedures. Thus, it is essential to choose an ML algorithm that can describe its purpose, rationale, and decision-making process that can be understood by both clinicians and patients; it can foster the confidence of mental health professionals in employing it to detect subjects with dementia quickly.

### Data limitation

**Fig. 14:**
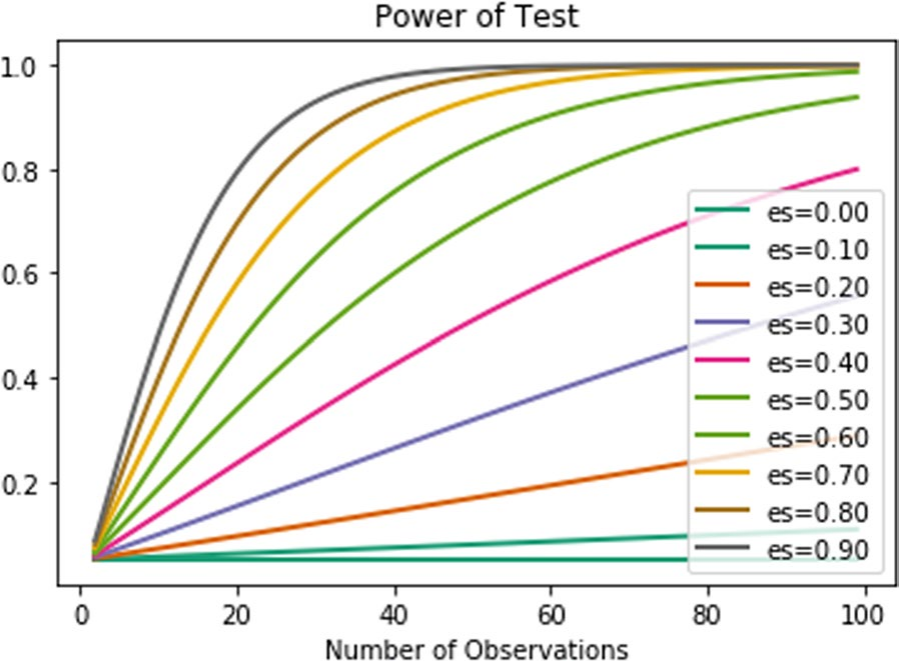
A description of using power analysis to estimate the minimum sample size is required for achieving a desired effect size; It shows the impact of different effect sizes (es) and various sizes of the data sample on the statistical power

No doubt having a lot of data samples, ML algorithms, which are cores of automatic SLAMs, can learn better [78] to map linguistic and acoustic features to the group of subjects (i.e., with dementia or without dementia). In other words, determining the optimal sample size for developing an efficient automatic SLAM assures adequate power to detect statistical significance [79]. However, for our problem, collecting language data from too many subjects is expensive and needs a lot of time. Thus, even it is necessary to estimate what is the sufficient size of samples for achieving acceptable classification results and then start to develop an automatic SLAM, but our results have shown that we could achieve good performance even by using the language data of less than 10 subjects (see Fig. 14).

## Conclusion

In this paper, we compared the performance of different ML classifiers with different types of features to assess dementia in older adults. Although this topic is widely explored in the literature, they rarely investigated how different language tasks, recording media, and modalities impact the performance of the classifiers. Our results showed that the classifiers that have been trained using the PDT dataset perform better than classifiers trained by the SRT dataset. We also found that the dataset obtained using phone-based recordings could increase ML classifiers’ performance compared to the web-based dataset. Finally, we showed that the classifiers trained only with the acoustic features had higher performance than classifiers trained with the linguistic features.

In the future, we will be working in the following directions: (1) Developing a cascade classifier that will be trained using both linguistic and acoustic features; (2) Using other types of data, such as eye-tracking; (3) Using few-shot ML algorithms and transfer learning techniques; (4) Considering pragmatic features such as fillers, GoAhead utterances, repetitions, incomplete words, and also contextual features using BERT (Bidirectional Encoder Representations from Transformers); and (5) Using text data augmentation techniques such as EDA: Easy Data Augmentation techniques to augment data samples.

## Data Availability

The datasets used and/or analyzed during the current study are available from the corresponding author on reasonable request.

## Footnotes

1 MCI refers to the condition where an older adult experiences cognitive impairment especially in tasks related to orientation and judgment [8].

2 It collected as a part of a project named TalkBank and has been done as a part of the Alzheimer Research Program at the University of Pittsburgh. The DB dataset was collected longitudinally, between 1983 and 1988, every year from around 200 patients with AD and 100 healthy controls.

3 It contains several subcorpora, generated according to neuropsychological tasks performed by the participants: (1) the Cookie Theft of the PDT (note that the participants’ speech related to this task have been transcribed), (2) the word (3) LFT, (4) the SRT, and (5) the sentence construction task.

4 Patients have been diagnosed by physicians from three hospitals in Toronto.

5 To collect speech data, the description of each task was provided for patients and healthy controls. Our trained examiners described tasks and provided them with examples to instruct them on how the task could be done.

6 All participants, who attended remotely, used phone line or web-interface to record their speeches. They asked to be in a place without background noise. The noisy speeches have been deleted.

7 Each subject has signed a consent form that was approved by the Research Ethics Board protocol 31127 of the University of Toronto.

8 Examiners explain to participants how they should complete language tasks. For example, for the PDT, they use the Cookie Theft picture or the Picnic Scene and tell participants describe everything they can see in this picture.

9 Thus, participants were shown a short passage with one of the following options (1) My Grandfather, (2) Rainbow or (3) Limpy.

10 People with dementia may use first person singular pronouns than physicians perhaps as a way of focusing attention on their perspective [56].

11 SA provides sentence embedding by averaging generated word embeddings from text files.

12 SIF provides sentence embedding by calculating the weighted average of word embeddings and removing their first principal component.

13 Coefficient of variation.
